# Quinacrine-Mediated Inhibition of Nrf2 Reverses Hypoxia-Induced 5-Fluorouracil Resistance in Colorectal Cancer

**DOI:** 10.3390/ijms20184366

**Published:** 2019-09-05

**Authors:** Ha Gyeong Kim, Chan Woo Kim, Don Haeng Lee, Jae-Seon Lee, Eun-Taex Oh, Heon Joo Park

**Affiliations:** 1 Department of Microbiology, College of Medicine, Inha University, Incheon 22212, Korea (H.G.K.) (C.W.K.); 2 Department of Internal Medicine, College of Medicine, Inha University, Incheon 22212, Korea; 3 Department of Molecular Medicine, College of Medicine, Inha University, Incheon 22212, Korea; 4 Hypoxia-related Disease Research Center, College of Medicine, Inha University, Incheon 22212, Korea; 5 Department of Biomedical Sciences, College of Medicine, Inha University, Incheon 22212, Korea

**Keywords:** quinacrine, 5-flourouracil, hypoxia, Nrf2, colorectal cancer

## Abstract

5-Fluorouracil (5-FU) is an important chemotherapeutic agent for the systemic treatment of colorectal cancer (CRC), but its effectiveness against CRC is limited by increased 5-FU resistance caused by the hypoxic tumor microenvironment. The purpose of our study was to assess the feasibility of using quinacrine (QC) to increase the efficacy of 5-FU against CRC cells under hypoxic conditions. QC reversed the resistance to 5-FU induced by hypoxia in CRC cell lines, as determined using ATP-Glo cell viability assays and clonogenic survival assays. Treatment of cells with 5-FU under hypoxic conditions had no effect on the expression of nuclear factor (erythroid-derived 2)-like 2 (Nrf2), a regulator of cellular resistance to oxidative stress, whereas treatment with QC alone or in combination with 5-FU reduced Nrf2 expression in all CRC cell lines tested. Overexpression of Nrf2 effectively prevented the increase in the number of DNA double-strand breaks induced by QC alone or in combination with 5-FU. siRNA-mediated c-Jun *N*-terminal kinase-1 (JNK1) knockdown inhibited the QC-mediated Nrf2 degradation in CRC cells under hypoxic conditions. The treatment of CRC xenografts in mice with the combination of QC and 5-FU was more effective in suppressing tumor growth than QC or 5-FU alone. QC increases the susceptibility of CRC cells to 5-FU under hypoxic conditions by enhancing JNK1-dependent Nrf2 degradation.

## 1. Introduction

Colorectal cancer (CRC) is one of the most prevalent and lethal tumor types in both men and women worldwide [1]. CRC patients frequently present with advanced and metastatic disease [2]. Although surgical resection of the primary tumor is the standard treatment for CRC [1], metastatic disease is generally considered to be untreatable. However, there are some exceptions in which resection is possible, including cases of oligometastatic lesions confined to the lung or liver [1,3]. Before surgical resection, patients are often administered a combination of cytotoxic chemotherapeutic agents, usually with targeted therapy [4]. Drugs commonly used for the treatment of CRC include 5-fluorouracil (5-FU), irinotecan, oxaliplatin, and leucovorin [4]. Among these drugs, 5-FU, which exerts its anticancer effects through the inhibition of thymidylate incorporation of metabolites into synthetic enzymes and DNA and RNA, is the most widely used agent for the systemic treatment of CRC [5]. Previously, 5-FU-mediated RpL3 (60S ribosomal protein L3) has been reported to promote p53-independent apoptosis in cancer cells [6,7]. Multiple clinical trials have demonstrated that single or combination therapy based on 5-FU yields a survival benefit for CRC patients [8].

For example, 10–15% of advanced CRC tumors treated with a first-line therapy of 5-FU/leucovorin showed some meaningful responses [4,5]; however, acquired resistance to 5-FU frequently develops and represents a serious clinical problem. A number of different mechanisms have been proposed to account for the resistance of CRC to 5-FU treatment. One such suggested resistance mechanism involves nuclear factor (erythroid-derived 2)-like 2 (Nrf2), which is overexpressed in a variety of cancers, including CRC [9,10,11,12,13,14,15]. 

Hypoxia and high oxidative stress, which are hallmarks of the tumor microenvironment [16], activate intracellular transcriptional processes that allow cancer cells to survive under these harsh conditions [17]. HIF-1 (hypoxia-inducible factor), the primary transcription factor responsible for mediating the cellular response to hypoxia, has been shown to promote the transcription of genes involved in angiogenesis, tumor growth, metastasis, metabolic reprogramming, chemoresistance, and radioresistance [16]. However, growing evidence indicates that the cellular response to hypoxia is much more complex, involving various signaling pathways, including (but not limited to) stress-response pathways [17]. Although it is not clear why hypoxia causes oxidative stress, hypoxia-induced oxidative stress activates Nrf2 signaling in cancer cells and initiates the expression of antioxidant-response genes, which in turn promote tumor survival and progression [17]. Therefore, the inhibition of Nrf2 signaling pathways may potentially improve the efficacy of treatment regimens by overcoming hypoxia-induced resistance.

Quinacrine (QC), a bioactive small-molecule derivative of 9-aminoacridine (9-AA), has historically been used clinically to treat malaria [18,19,20] but continues to be used to treat tapeworm infestations, connective tissue diseases, and giardiasis [18,19,20]. Chronic daily use of QC has been reported to cause certain side effects, such as gastrointestinal upset and the yellowing of the sclera and skin, in a small percentage of patients [21]. For the treatment of malaria, QC has been replaced by chloroquine because the latter drug is both more effective and less toxic [21]. QC has recently attracted renewed interest as an anticancer agent because it targets several major cancer-promoting pathways in various cancers (e.g., colon, breast, pancreatic, lung, and renal cell carcinoma) while causing minimal undesirable side effects in normal tissues [20,21,22,23,24]. It has been shown that the anticancer effects of QC are closely related to the inhibition of the PI3K (phosphatidylinositol-3-kinase)/AKT (protein kinase B)/mTOR (mammalian target of rapamycin) and NF-κB (nuclear factor kappa light chain enhancer of activated B cells) signaling pathways and to the activation of p53 in cancer cells [22]. Although using QC alone has not shown promising results in the treatment of cancer [25], recent preclinical studies have provided evidence that QC may enhance the efficacy of other anticancer drugs, including suberoylanilide hydroxamic acid (SAHA), sorafenib, and 5-FU [25,26,27]. In this regard, QC has been shown to potentiate the apoptosis of cancer cells induced by chemotherapeutic agents [18,19,20,27,28,29]. However, how the hypoxic tumor microenvironment might impact the ability of QC to enhance the efficacy of chemotherapy has not been fully elucidated.

Here, we report that QC combines favorably with 5-FU in an additive-to-synergistic manner against CRC cells under hypoxic conditions. Specifically, we found that QC effectively increases the cytotoxicity of 5-FU towards CRC cells under hypoxic conditions in vitro and in vivo by inhibiting Nrf2. In addition, we showed that QC inhibits Nrf2 through the activation of c-Jun *N*-terminal kinase-1 (JNK1) in CRC cells in hypoxia. Collectively, these findings provide a rationale for future clinical trials of the drug combination QC/5-FU for the treatment of CRC. 

## 2. Results

### 2.1. QC Sensitizes CRC Cells to 5-FU in Hypoxia

**Figure 1:**
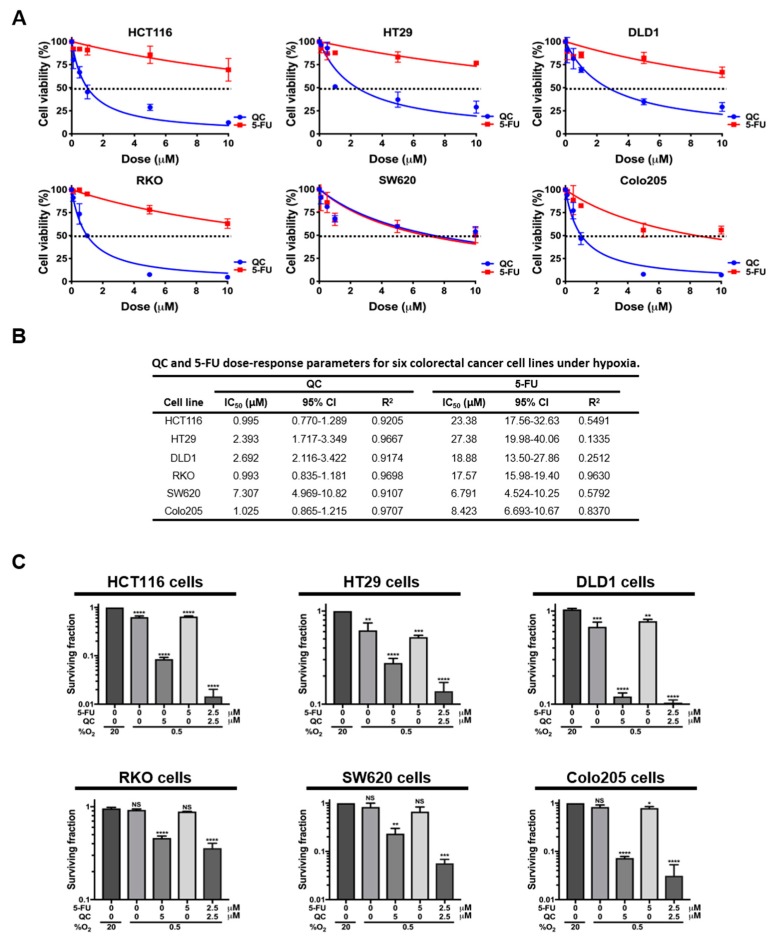
Quinacrine (QC) sensitizes colorectal cancer (CRC) cells to 5-fluorouracil (5-FU) treatment under hypoxic conditions. (**A**) ATP-Glo assay of the QC or 5-FU treatments in HCT116, HT29, DLD1, RKO, SW620, and Colo205 cells. (**B**) Summary of IC_50_ values and associated 95% confidence intervals (CI) for QC, 5-FU, and combined QC and 5-FU treatment in all tested CRC cell lines. (**C**) Clonogenic survival assay for the QC/5-FU combination and single-agent treatments in HCT116, HT29, DLD1, RKO, SW620, and Colo205 cells. Data are presented as means ± SD (* *p* < 0.05, ** *p* < 0.01, *** *p* < 0.001, **** *p* < 0.0001 by ANOVA). “NS” indicates not significant (*p* > 0.05).

Recent studies have demonstrated that QC may enhance the efficacy of other anticancer drugs, including SAHA, sorafenib, and 5-FU [25,26,27]. In addition, QC synergizes with 5-FU in CRC cells in normoxia [29]. Therefore, we investigated whether QC synergizes with 5-FU in CRC cells in hypoxia. First, we assessed the effect of QC and 5-FU on cancer cell death in all CRC models (HCT116, HT29, DLD1, RKO, SW620, and Colo205 cells) using an ATP (adenosine tri-phosphate)-Glo cell viability assay. In normoxia (20% O_2_), both QC and 5-FU increased cell death in all tested CRC models (Figure S1A). Half-maximal inhibitory concentration (IC_50_) values of QC and 5-FU for CRC cells are summarized in Figure S1B. As shown in Figure 1A, the cytotoxic effect of 5-FU on all tested CRC cells was significantly reduced in hypoxia, whereas the cytotoxicity of QC in hypoxia was similar to that in normoxia (Figure S1A and Figure 1A). IC_50_ values of QC and 5-FU for CRC cells under hypoxia are summarized in Figure 1B. To assess whether QC synergizes with 5-FU in CRC cells in normoxia and hypoxia, we performed clonogenic survival assays in HCT116, HT29, DLD1, RKO, SW620, and Colo205 cells following treatment with QC, 5-FU, or both QC and 5-FU. In normoxia, the administration of each single drug or the combination of QC and 5-FU increased the clonogenic cell death of CRC cells (Figure S2C); while QC also induced clonogenic cell death in CRC cells in hypoxia, 5-FU was less effective in hypoxia than in normoxia at inducing clonogenic cell death in CRC cells (Figure 1C). Importantly, under hypoxic conditions, the incubation of cells with 5-FU together with an equimolar concentration of QC was far more effective than 5-FU alone, demonstrating that QC sensitized CRC cells to 5-FU (Figure 1C). Notably, similar results were obtained for other types of cancer cell lines, including MDA-MB-231 (breast), U87-MG (brain), and MIA-PaCa-2 (pancreas) (Figure S2A–E). Taken together, these findings indicate that QC synergizes with 5-FU in CRC cells and sensitizes them to 5-FU in hypoxia.

### 2.2. QC Sensitizes CRC Cells to 5-FU under Hypoxic Conditions by Inhibiting Nrf2

**Figure 2:**
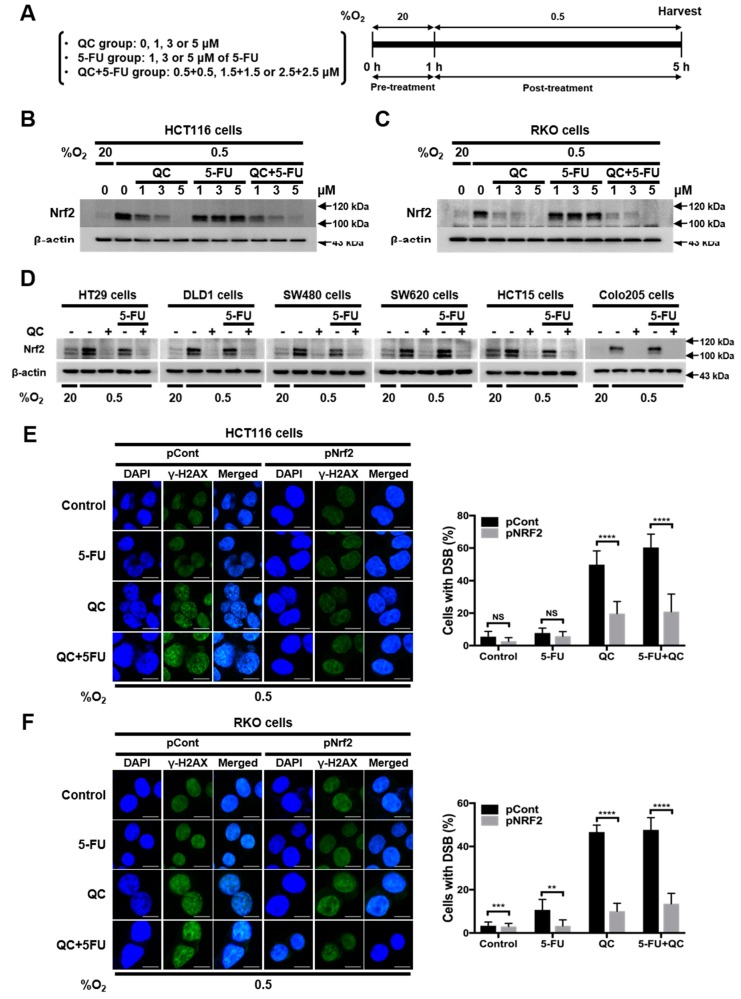
QC sensitizes CRC cells to 5-FU in hypoxia by inhibiting nuclear factor (erythroid-derived 2)-like 2 (Nrf2). (**A**) Scheme of the treatment and sampling procedure. (**B,C**) Effect of the QC/5-FU combination on Nrf2 expression in HCT116 (**B**) and RKO (**C**) cells under hypoxic conditions. (**D**) Effect of the QC/5-FU combination on Nrf2 expression in HT-29, DLD1, SW480, SW620, HCT15, and Colo205 cells under hypoxic conditions. Representative images of Nrf2 and β-actin were detected by immunoblot. Representative images of immunoblots for each protein were obtained using the same sample on different gels after a single experiment. (**E,F**) Effect of Nrf2 overexpression on DNA damage induced in HCT116 (**E**) and RKO (**F**) cells by the QC/5-FU combination under hypoxic conditions. γ-H2AX (green) staining in HCT116 (**E**) and RKO (**F**) cells following treatment (left), and the relative percentage of foci-positive cells (right). Data are presented as means ± SD (** *p* < 0.01, *** *p* < 0.001, **** *p* < 0.0001 by ANOVA). “NS” indicates not significant (*p* > 0.05).

Next, we investigated whether QC increases the sensitivity of CRC cells to 5-FU in hypoxia by inhibiting Nrf2 expression. The exposure of HCT116 and RKO cells to hypoxia for 8 h induced a marked increase in Nrf2 expression, which peaked at 4 h (Figure S3A,B). A previous report identified QC as an Nrf2 inhibitor [30]. To determine the effects of QC and 5-FU, alone and in combination, on Nrf2 expression in CRC cells, we incubated HCT116 and RKO cells with each drug alone and with an equimolar combination of QC (0–5 μM) and 5-FU (0–5 μM) for 1 h in normoxia. We then exposed the cells to hypoxia for 4 h and assessed Nrf2 expression by immunoblot analysis. Treatment with 5-FU alone did not induce significant changes in Nrf2 expression in CRC cells under hypoxic conditions (Figure 2B,C). In contrast, the treatment of cells under hypoxic conditions with QC alone or in combination with 5-FU caused a dose-dependent decrease in Nrf2 expression (Figure 2B,C). Similar results were observed in HT29, DLD1, SW480, SW620, HCT15, and Colo205 CRC cells (Figure 2D). To confirm that QC sensitizes CRC cells to 5-FU in hypoxia by inhibiting Nrf2, we performed an Nrf2 gain-of-function experiment. Previous reports demonstrated that QC and 5-FU cause cDNA damage [31], which can be detected by monitoring H2AX (H2A histone family member X), a marker of DNA double-stand breakage (DSB) and the activation of the DNA-damage response [25]. The treatment of cells with QC (5 μM) or the combination of QC (2.5 μM) and 5-FU (2.5 μM) under hypoxic conditions increased DSB levels in HCT116 and RKO cells, whereas 5-FU alone did not (Figure 2E,F). Notably, the overexpression of Nrf2 attenuated the ability of QC, alone or in combination with 5-FU, to suppress Nrf2 in HCT116 and RKO cells in hypoxia (Figure S4), as evidenced by a reduction in DSBs under these conditions (Figure 2E,F). Collectively, these results provide evidence that QC sensitizes CRC cells to 5-FU by inhibiting Nrf2 in hypoxia. 

### 2.3. QC Decreases the Stability of Nrf2 Protein

**Figure 3:**
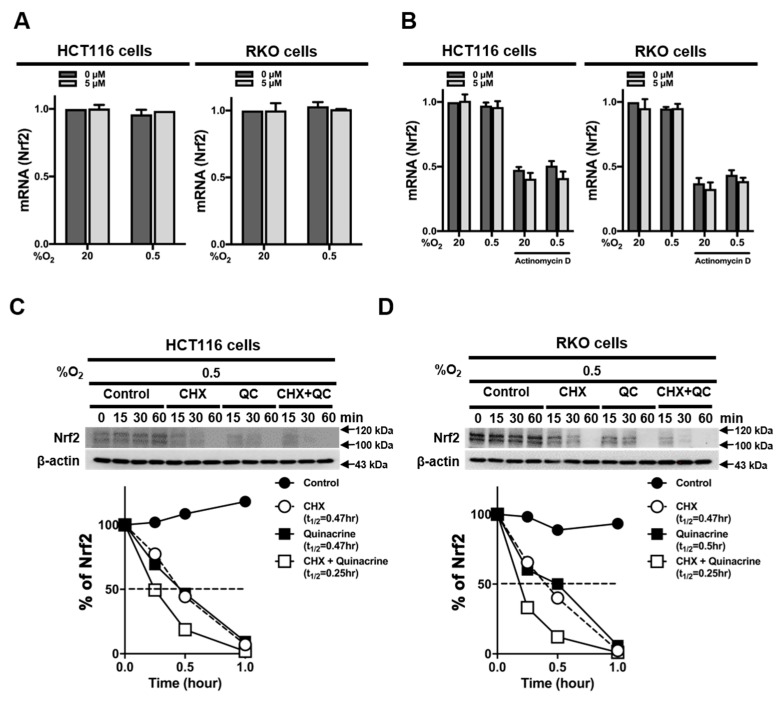
QC decreases Nrf2 protein stability in CRC cells. (**A,B**) Effects of QC on *Nrf2* mRNA expression (**A**) and *Nrf2* mRNA stability (**B**) in HCT116 cells (left) and RKO cells (right). (**C,D**) Effect of QC on Nrf2 protein stability in HCT116 cells (**C**) and RKO cells (**D**) under hypoxic conditions. Nrf2 protein levels were quantified using Image J software; band intensities were normalized to those of β-actin (band intensity at t_0_ was defined as 100%). Representative images of Nrf2 and β-actin were detected by immunoblot. Representative images of immunoblots for each protein were obtained using the same sample on different gels after a single experiment.

To determine how QC inhibits Nrf2 expression in CRC cells, we first measured expression levels of *Nrf2* mRNA in the absence or presence of QC in normoxia and hypoxia. We found that QC did not alter *Nrf2* mRNA expression in CRC cells, regardless of O_2_ tension (Figure 3A). To determine whether QC affects the mRNA stability of Nrf2 in CRC cells, we treated HCT116 and RKO cells with 5 μM QC under normoxia or hypoxia for 4 h in the presence of 5 μg/mL actinomycin D, which blocks de novo mRNA synthesis. QC did not alter *Nrf2* mRNA expression in either of these cell lines under normoxia or hypoxia (Figure 3B), suggesting that QC does not regulate the transcription or degradation of *Nrf2* mRNA. To test whether QC affects the accumulation and stability of Nrf2 protein, we incubated HCT116 and RKO cells in hypoxia for 1 h with QC in the presence of the protein synthesis inhibitor cycloheximide (CHX) and monitored decreases in Nrf2 protein levels over time in its absence by immunoblotting. Under hypoxic conditions in the presence of 10 μg/mL CHX, 5 μM QC decreased the half-life of Nrf2 from 0.47 to 0.25 h (Figure 3C,D), suggesting that QC decreases Nrf2 expression by decreasing the stability of the Nrf2 protein.

### 2.4. JNK1 Activation is Required for QC-Mediated Degradation of Nrf2 Protein

**Figure 4:**
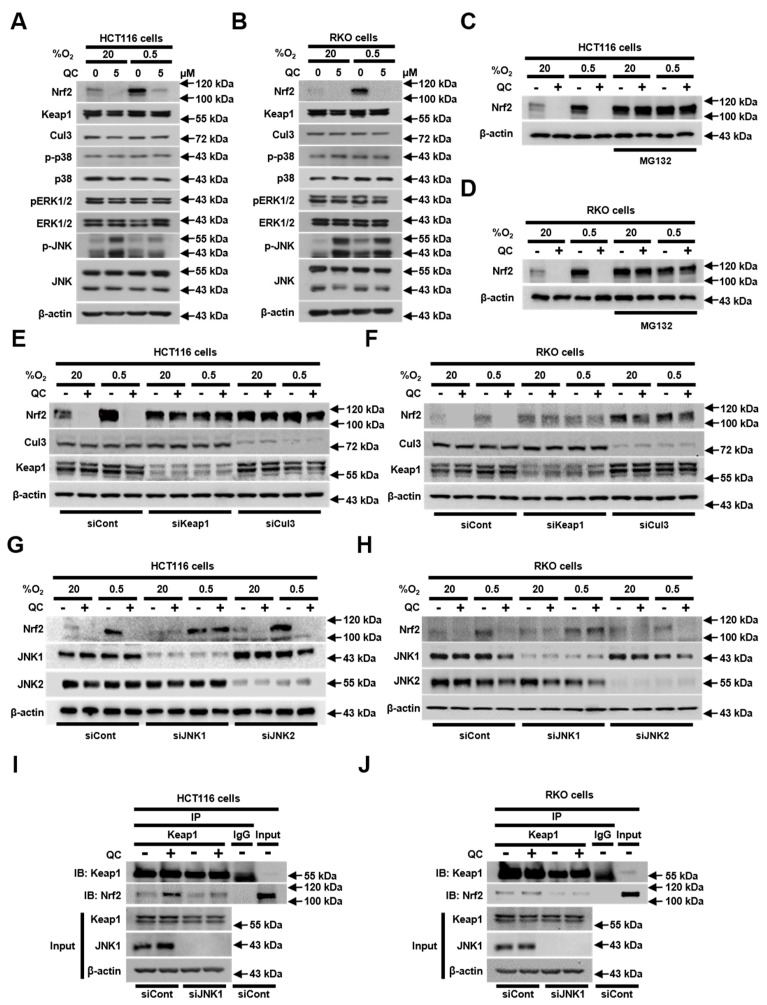
JNK1 activation is required for the QC-induced degradation of Nrf2 protein. (**A,B**) Effect of QC on the expression of Nrf2, Keap1, Cul3, p-p38, p38, pERK1/2 (phospho extracellular signal-regulated kinases), ERK1/2, p-JNK, JNK, and β-actin in HCT116 cells (**A**) and RKO cells (**B**) under normoxic and hypoxic conditions. (**C,D**) Effect of QC on the proteasome-mediated degradation of Nrf2 in HCT116 cells (**C**) and RKO cells (**D**) under normoxic and hypoxic conditions. (**E,F**) Effect of QC on Keap1/Cul3-dependent degradation of Nrf2 in HCT116 cells (**E**) and RKO cells (**F**) under normoxic and hypoxic conditions. (**G,H**) Effect of JNK1 activation on QC-induced inhibition of Nrf2 in HCT116 cells (**G**) and RKO cells (**H**) under normoxic and hypoxic conditions. (**I,J**) Effect of QC on the interaction between Nrf2 and Keap1 in HCT116 cells (**I**) and RKO cells (**J**) under hypoxic conditions. Representative images of Nrf2, Keap1, Cul3, p-p38, p38, pERK1/2, ERK1/2, pJNK, JNK, and β-actin were detected by immunoblot. Representative images of immunoblots for each protein were obtained using the same sample on different gels after a single experiment.

Previous reports have shown that Keap1 (kelch-like enoyl-CoA hydratase-associated protein) continuously facilitates ubiquitination-dependent, proteasome-mediated Nrf2 degradation by recruiting the Cul3/RBX (ring-box protein) E3 ubiquitin ligase complex [32]. In addition, it has been reported that mitogen-activated protein kinases (MAPKs) regulate Nrf2 signaling [33]. Accordingly, we investigated whether QC affects the expression levels of Keap1, Cul3 (cullin 3) and MAPKs. As shown in Figure 4A,B, QC did not alter the levels of these proteins in CRC cells, regardless of the oxygenation status. However, QC treatment did increase the phosphorylation (activation) of JNKs in both normoxia and hypoxia (Figure 4A,B). To determine whether QC increases the proteasome-mediated degradation of Nrf2, we pretreated HCT116 and RKO cells with MG132 (a proteasome inhibitor) for 1 h. We then treated cells with 5 μM QC in normoxia or hypoxia for 4 h, after which we assessed Nrf2 protein levels by immunoblotting. As shown in Figure 4C,D, MG132 pretreatment inhibited the QC-induced degradation of Nrf2 in CRC cells in both normoxia and hypoxia. To determine whether this QC-induced increase in the degradation of Nrf2 is mediated by Keap1 and the Cul3/RBX E3 ubiquitin ligase complex, we transfected HCT116 and RKO cells with small interfering RNAs (siRNAs) targeting Keap1 or Cul3. Both siKeap1 and siCul3 efficiently prevented the QC-induced degradation of Nrf2 (Figure 4E,F). To define the potential contributions of individual JNKs to the regulation of Nrf2 in QC-treated cells under normoxic and hypoxic conditions, we transfected HCT116 and RKO cells with siRNAs targeting JNK1 or JNK2, and then incubated cells with or without 5 μM QC in normoxia or hypoxia for 4 h. siJNK1 significantly inhibited the QC-induced degradation of Nrf2 in CRC cells under both normoxic and hypoxic conditions (Figure 4G,H). Since it has been reported that Nrf2 activity is negatively regulated by Keap1 [32], we investigated whether JNK1 affects the Keap1-dependent degradation of Nrf2 in QC-treated CRC cells. To this end, we treated MG132-pretreated HCT116 and RKO cells with 5 μM QC and assessed the interaction between endogenous Nrf2 and Keap1 using co-immunoprecipitation and immunoblot analyses. As shown in Figure 4I,J, QC enhanced the interaction between Nrf2 and Keap1 in CRC cells, and siJNK reduced this interaction, regardless of QC treatment. These results suggest that JNK1 contributes to the degradation of Nrf2 by promoting the interaction between Keap1 and Nrf2 in QC-treated CRC cells.

### 2.5. QC Inhibits Tumor Growth In Vivo”

**Figure 5:**
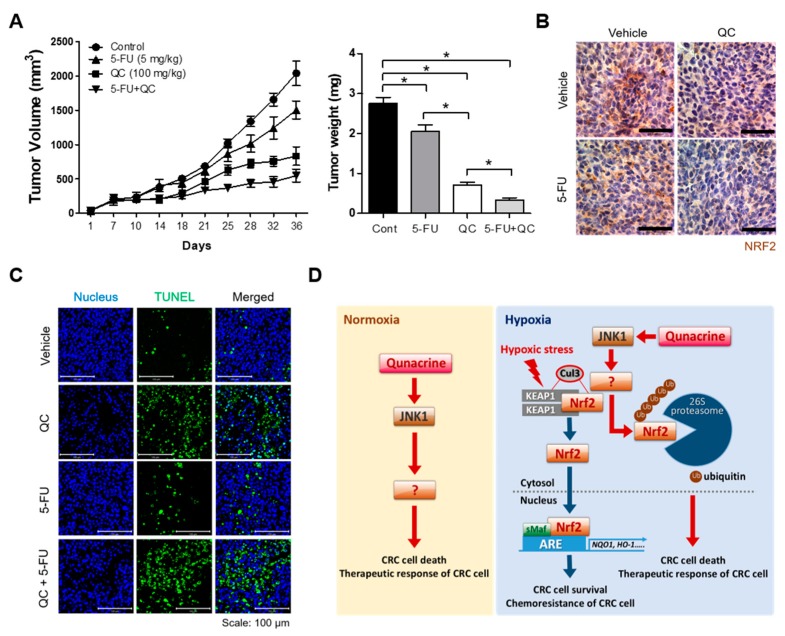
The QC/5-FU combination inhibits tumor growth in a tumor xenograft mouse model. (**A**) Tumor volume was measured at the indicated times (left panel), and tumor weight was quantified at the end of the experiment (right panel). Data are presented as means ± SEM (*n* = 7 mice/group; * *p* < 0.05). (**B**) Representative images of immunohistochemical analyses of Nrf2 in tumors derived from HCT116 xenografts. Scale bar = 50 μm. (**C**) Apoptotic cells were detected in HCT116 xenograft tumors using a TUNEL (terminal deoxynucleotidyl transferase dUTP nick end labeling) assay kit. Scale bar = 100 μm. Blue, nuclei; green, TUNEL staining. (**D**) Schematic model showing how QC reverses hypoxia-induced 5-FU resistance in CRC cells under normoxia and hypoxia by inhibiting Nrf2.

To evaluate the 5-FU–sensitizing action of QC in vivo, we assessed the antitumor effects of 5-FU, alone and in combination with QC, in an HCT116 mouse xenograft model, prepared as described in Section 4. When tumor volumes reached ~100 mm^3^, we administered QC (100 mg/kg) by oral gavage and 5-FU (5 mg/kg) by intraperitoneal (IP) injection three times a week. Tumor size was measured every 3 to 4 days for 36 days. All mice were sacrificed at the end of the experiment, and tumors were dissected and weighed. As shown in Figure 5A, treatment with QC or 5-FU alone significantly suppressed the growth of HCT116 xenografts, reducing the tumor volume from 2039.82 ± 438.82 in the absence of treatment (vehicle control) to 833.67 ± 133.19 mm^3^ (100 mg/kg QC) and 1504.5 ± 128.64 (5 mg/kg 5-FU) (*p* < 0.05). Combined administration of QC and 5-FU at the same doses exerted a significant inhibitor effect, reducing the tumor volume to 548.28 ± 224.38 mm^3^ (*p < 0.05; Figure 5A). Consistent with these results, HCT116 tumor weights were ~0.74-, 0.26-, and 0.852-fold lower in mice treated with QC, 5-FU, or the QC/5-FU combination, respectively, compared with vehicle-treated mice (Figure 5A). Using immunohistochemical staining, we further found that QC induced Nrf2 degradation in vivo, as evidenced by significantly decreased Nrf2 expression in tumor tissues. However, 5-FU treatment alone did not affect Nrf2 expression in HCT116 xenografts (Figure 5B). Finally, we evaluated the effect of QC on Nrf2 degradation and cell death in CRC cells in vivo. QC treatment increased apoptotic bodies in tumor tissues (Figure 5C). Taken together, our results indicate that QC induces CRC cell death by promoting the JNK1-dependent, proteasome-mediated degradation of Nrf2 in tumors.

## 3. Discussion

We found that the combination of QC and 5-FU effectively induces cancer cell death under hypoxic conditions in preclinical CRC models by inhibiting Nrf2. We further found that QC activates JNK1, leading to the inhibition of Nrf2 expression, thereby causing cancer cell death under hypoxic conditions in vitro and suppressing tumor growth in an in vivo model. In addition, our results demonstrate that QC-induced JNK1 activation increases the interaction between Keap1 and Nrf2.

CRC remains a significant cause of mortality and morbidity worldwide [1]. Notably, CRC patients frequently present with progressive metastatic disease [2]. Currently, neoadjuvant chemotherapy is used for the treatment of synchronous or metachronous colorectal liver metastases before hepatic surgery [4]. 5-FU is an important chemotherapeutic agent for the systemic treatment of CRC, and combinations of 5-FU with various anticancer drugs are being tested as first-line therapies for advanced CRC prior to surgery [4,5]. Although it has been shown that the response of advanced CRC to treatment is limited, multiple clinical trials have demonstrated that single or combination therapy based on 5-FU affords a survival benefit for CRC patients [8]. 

However, it has been reported that the hypoxic tumor microenvironment increases 5-FU resistance, resulting in poor responses of CRC patients to 5-FU–based chemotherapy [10]. Nrf2 overexpression induced by hypoxia and high oxidative stress in the tumor microenvironment is correlated with increased 5-FU resistance in CRC patients [10]. 

Hypoxia and high oxidative status resulting from an inadequate and poorly formed vasculature are common characteristics of the tumor microenvironment [34]. In such a tumor microenvironment, hypoxia-induced oxidative stress activates Nrf2 signaling in cancer cells, thereby initiating the expression of antioxidant response genes, which promote tumor survival and progression [17]. In human cancers, genetic and epigenetic alterations lead to the constitutive high-level expression of Nrf2 [17,28,29,35], which protects cancer cells from the excessive oxidative stress caused by chemotherapies and radiotherapies [33,34]. Previous reports have demonstrated that Nrf2 increases HIF-1α–mediated angiogenesis in colon tumors and the migration of esophageal squamous cell carcinoma in the hypoxic tumor microenvironment [36,37]. Furthermore, it has been reported that HIF-1α increases 5-FU resistance in cancer cells in hypoxia [38]. Previously, RpL3 has been reported to be one of the major molecules of 5-FU resistance in p53-mutated cancer cells by controlling cellular redox status independent of Nrf2 [39]. Given the complexity of 5-FU resistance mechanisms in CRC under hypoxic conditions, the use of combined therapy may be an effective means of overcoming the limited clinical efficacy of current therapeutic strategies.

QC, used historically as an antimalarial drug [18,19,20], has recently attracted research attention as an anticancer agent that targets major cancer-promoting pathways in various cancers (e.g., colon, breast, pancreatic, lung, and renal cell carcinoma) while minimally affecting normal tissues [20,21,22,23,24]. A previous report demonstrated that QC targets PI3K/AKT/mTOR, NF-kB and p53 signaling pathways [22]. However, QC alone may not be an effective cancer treatment agent [25]. Some preclinical studies have reported that QC may be a chemosensitizer capable of increasing the apoptotic responses of cancer cells to chemotherapeutic agents [25,26], such as 5-FU, SAHA, and sorafenib [25,26,27]. Previous reports have also identified QC as an Nrf2 inhibitor, but the underlying molecular mechanism has not been described [30]. In agreement with this latter report, our results showed that QC effectively inhibited hypoxia-induced Nrf2 expression in CRC cells (Figure 2A–C). We further found that the QC-mediated inhibition of Nrf2 sensitizes CRC cells to 5-FU, which has been widely used in the treatment of breast, gastric and pancreatic cancers, as well as squamous cell carcinomas arising in the head and neck [8] under hypoxic conditions (Figure 1 and Figure 2). Our investigation yielded similar results, showing that QC increases the sensitivity of CRC cells to 5-FU in hypoxia (Figure S2A–E). As shown in Figure S4 and Figure 2D,E, the overexpression of Nrf2 attenuated the decrease in Nrf2 expression and DSBs in CRC cells caused by QC alone or in combination with 5-FU. It has been reported that QC synergizes with 5-FU in CRC cells under normoxia [29]. However, the mechanism by which QC increases the sensitivity of CRC cells to 5-FU and induces cancer cell death under normoxia is unclear. In agreement with the report, we found that QC increases the sensitivity of CRC cells to 5-FU in normoxia (Figures S1 and S2). In addition, we further found that QC activates JNKs in CRC cells under normoxia (Figure 4A,B). Therefore, additional studies will be required to explore the mechanism by which QC increases the sensitivity of CRC cells to 5-FU under normoxia. In summary, our results indicate that QC-induced inhibition of Nrf2 and increased sensitivity of CRC cells to 5-FU causes increased cancer cell death under hypoxic conditions and suppresses the growth of xenograft tumors (Figure 5).

Keap1 is known to act through the recruitment of the Cul3/RBX1 E3 ubiquitin ligase complex and subsequent proteasome-mediated degradation to regulate Nrf2 protein stability and thereby contribute to maintaining a low level of Nrf2 in the cell [40,41,42]. Because the mitogen-activated protein kinase (MAPK) cascade has been shown to play an important role in the intracellular signaling of hypoxic cancer cells [34] and to regulate the Nrf2 signaling pathway [33], we investigated whether QC regulates MAPKs, as well as Keap1 and Cul3, in CRC cells under either normoxic or hypoxic conditions. As shown in Figure 4A,B, QC did not alter the levels of the tested proteins but did induce the phosphorylation of JNKs. Using specific siRNAs to investigate the potential role of JNKs in mediating the effects of QC in HCT116 and RKO cells, we found that siJNK1 restored Nrf2 degradation in QC-treated cells (Figure 4). A previous report demonstrated that MAPKs regulate Nrf2 signaling, thereby increasing Nrf2 phosphorylation [33]. Therefore, we hypothesized that the QC-induced activation of JNK1 indirectly downregulates Nrf2. However, additional studies will be required to explore the mechanism by which QC-induced JNK1 phosphorylation downregulates Nrf2 (Figure 5D). We subsequently investigated how JNK1 affects the Keap1-dependent degradation of Nrf2 in QC-treated cancer cells, demonstrating that QC increased the interaction between Nrf2 and Keap1 and that siJNK decreased this interaction in the presence or absence of QC (Figure 4I,J). Thus, our results suggest that the QC-induced activation of JNK1 contributes to Nrf2 degradation in QC-treated CRC cells by promoting the interaction between Keap1 and Nrf2 (Figure 4). 

In conclusion, our findings demonstrate that QC effectively enhances the cytotoxicity of 5-FU toward CRC cells in hypoxia (Figure 5D), and we further show that QC reverses hypoxia-induced 5-FU resistance in CRC cells by inhibiting Nrf2 through activation of JNK1. In addition, our findings provide a rationale for potential clinical trials of the drug combination, QC and 5-FU, for CRC management.

## 4. Materials and Methods

### 4.1. Cell Lines and Culture Conditions 

The human colorectal cancer cell lines (HCT116, HT29, DLD1, RKO, SW480, SW620, HCT15, Colo205, and SW480), the human breast cancer cell line (MDA-MB-231), the human pancreatic cancer cell line (MIA-PaCa-2), and the human glioma cell line (U87-MG) were obtained from American Type Culture Collection (ATCC, Manassas, VA, USA) and cultured in DMEM (Invitrogen, Camarillo, CA, USA) or RPMI (Roswell Park Memorial Institute) (Invitrogen, Camarillo, CA, USA). Cells were incubated at 37 °C in a 5% CO_2_-containing humidified incubator unless otherwise noted. Treatments of cancer cells with various drugs in hypoxic or normoxic conditions were performed in an InvivO_2_ 500 hypoxia workstation (The Baker Company, Sanford, ME, USA) gassed with a mixture of 0.5% oxygen or 20% oxygen, 5% CO_2_ and balanced with nitrogen. 

### 4.2. Chemicals and Antibodies

QC and 5-FU were purchased from Sigma-Aldrich (St. Louis, MO, USA). CHX (cycloheximide) and MG132 were purchased from Calbiochem (Darmstadt, Hesse, Germany). Primary antibodies against the following proteins were used: Nrf2, phospho-p38, p38, phospho-ERK, ERK, phospho-JNK, JNK, Keap1, Cul3 (all from Cell Signaling Technology, Beverly, MA, USA), β-actin (Sigma-Aldrich, St. Louis, MO, USA), phospho-γH2AX (Millipore, Darmstadt, Hesse, Germany) and HIF-1α (R&D Systems, Minneapolis, MN, USA). The secondary antibodies used for immunoblotting included horseradish peroxidase (HRP)-conjugated anti-mouse and anti-rabbit (Cell Signaling Technology, Beverly, MA, USA).

### 4.3. Quantification of Clonogenic Cell Death

Varying numbers of cells were plated on 60 mm dishes and treated with various doses of QC or 5-FU under normoxia or hypoxia for 4 h. Cells were then incubated in drug-free medium for 14 days at 37 °C in a 5% CO_2_ incubator to allow colony formation. The culture medium was decanted, and the colonies were fixed with 95% methanol and stained with 0.5% crystal violet. The numbers of colonies (>50 cells) from triplicate dishes or plates were counted, and the mean numbers of colonies formed by drug-treated cells were compared with those formed by untreated cells.

### 4.4. ATP-Glo Cell Viability Assay

Cells (1000 cells/well) were seeded in 96-well microplates, and various concentrations of QC (0–10 μM) or 5-FU (0–10 μM) were administered. After 24 h, quantitative estimations of cell growth and survival (IC50) were determined using the cell Titer AT

P-Glo Cell Viability Assay (Promega, Madison, WI, USA). Measurements using a Luminometer (Perkin Elmer, Meriden, CT, USA) were conducted following the manufacture’s protocol. 

### 4.5. DNA Double-Strand Break Staining

Cells were seeded in an eight-well chamber slide (SPL Life Sciences, Pocheon-si, Republic of Korea) and incubated overnight in a humidified 5% CO_2_/95% air incubator at 37 °C. After treating with QC (0–5 μM) or 5-FU (0–5 μM) for 12 h, the cells were fixed with 3.7% (*v/v*) PFA for 15 min, washed three times with PBS (phosphate-buffered saline), permeabilized with 0.25% (*v/v*) Triton X-100 and washed three times with PBS. Then, the cells were blocked for 30 min at room temperature with PBS containing 3% (*w/v*) bovine serum albumin. The cells were treated with anti-γ-H2AX antibody solution and incubated overnight at 4 °C. The cells were washed three times with PBS and incubated secondary antibody-FITC (Thermo Fisher Scientific, Pittsburh, PA, USA) conjugate solution for 1 h and washed three times with PBS. The cell nuclei were stained with DAPI (Sigma-Aldrich, St. Louis, MO, USA) for 2 min. The cells were washed three times with PBS, and the slides were mounted with mounting medium and cover slides. The fluorescence image of γ-H2AX foci was performed with a Nikon C1-Plus laser-scanning TE200E confocal microscope (Nikon, Tokyo, Japan), and the γ-H2AX foci were analyzed with image J software (NIH, Bethesda, MD, USA)

### 4.6. Immunoblot Analysis and Immunoprecipitation

Cells were lysed using ice-cold RIPA (radioimmunoprecipitation assay buffer) buffer containing an inhibitor cocktail (Roche Applied Science, Indianapolis, IN, USA), sodium orthovanadate (Sigma-Aldrich, St. Louis, MO, USA), and sodium fluoride (Sigma-Aldrich, St. Louis, MO, USA). The lysates were resolved by SDS-PAGE and subjected to immunoblot analysis. Signals were detected by chemiluminescence (Pierce, Rockford, IL, USA). For immunoprecipitation, cells were lysed using a lysis buffer (10 mM Tris-HCl pH 7.5, 150 mM NaCl, 1 mM EDTA, 0.1% NP-40, 1 mM PMSF (phenylmethylsulfonyl fluoride)) containing an inhibitor cocktail (Roche Applied Science, Indianapolis, IN, USA), sodium orthovanadate, and sodium fluoride. One milligram of total protein was precipitated using 1 μg of anti-Keap1 antibody and collected with Protein G-magnetic beads (New England Biolabs, Beverly, MA, USA) at 4 °C for 16 h. The immunoprecipitate was washed four times with cold lysis buffer, and bound proteins were resolved by SDS-PAGE and analyzed by immunoblot analysis. The chemiluminescent signals of membranes were detected by ChemiDoc^TM^ Touch Imaging System (Bio-Rad Laboratoreis, Inc, Hercules, CA, USA) and analyzed by Image Lab^TM^ Version 6.0.0 build 25 Standard Edition (Bio-Rad Laboratoreis, Inc, Hercules, CA, USA).

### 4.7. siRNA Transfection

Keap1, Cul3, JNK1 and JNK2 were knocked down by RNA interference (RNAi) using the following 19-bp (including a 2-deoxynucleotide overhang) siRNAs (Bioneer, Daejeon, Republic of Korea): Keap1, CAGAUUGACCAGCAGAACUdTdT; Cul3, AGGUCUCCUGAAUACCUdTdT; JNK1, AAGCCCAGUAAUAUAGUAGUAdTdT; and JNK2, CUGUAACUGUUGAGAUGUATTdTdT. Stealth RNAi (Invitrogen, Camarillo, CA, USA) was used as a negative control (siCont). For transfection, cells were seeded to 25 cm^2^ flasks, grown to ~80% confluence, and transfected with siRNA duplexes using LipofectAMINE 2000 (Invitrogen, Camarillo, CA, USA) according to the manufacturer’s recommendations. After incubation for 48 h, the cells were processed as indicated for each analysis.

### 4.8. Plasmid Construction

To construct plasmids expressing Nrf2 protein, total RNA was obtained from RKO cells using the TRIzol reagent (Invitrogen, Camarillo, CA, USA) and cDNA was generated using SuperScript^TM^III Reverse Transcriptase (Invitrogen, Camarillo, CA, USA). The open reading frame (ORF) of Nrf2 was PCR amplified with appropriate primers, and the PCR products were digested with restriction enzymes and directly ligated into the pCDNA3.1 (Invitrogen, Camarillo, CA, USA) vector for cloning. The cloned plasmids were analyzed by restriction digestion and DNA sequencing (Bionics, Seoul, Korea). 

### 4.9. Tumor Xenograft Experiments

All animal experiments were carried out according to the Institutional Animal Care and Use Committee protocol approved by Inha University (INHA 150605-383). Male 8-week-old nude mice (BALB/c-nu) were purchased from Orient Bio Laboratory Animal Inc. (Seoul, Republic of Korea) and maintained under a 12 h light/12 h dark cycle with food and water provided ad libitum. HCT116 cells (5 × 10^6^) were injected subcutaneously into the right flanks. Tumor size was measured with calipers, and mice were divided into homogeneous cohorts according to their initial tumor volume. The mice were treated with quinacrine alone (100 mg/kg, orally, three times a week), 5-FU alone (5 mg/kg, intraperitoneal injection, three times a week), or quinacrine (100 mg/kg, orally, three times a week) in combination with 5-FU (5 mg/kg, intraperitoneal injection, three times a week) for 36 days. Tumor volume was calculated every 3 to 4 days with a caliper, using the following formula: volume = [length × (width)^2^]/2. 

### 4.10. Immunohistochemistry

Immunohistochemical analysis of Nrf2 was performed with a Vectastain Elite ABC kit (Vector Laboratories Inc., Burlingame, CA, USA) following the manufacturer’s protocol. For antigen retrieval, sections were placed in citrate buffer (pH 6.0) and heated in a microwave oven for 10 min. For immunoperoxidase labeling, endogenous peroxidase was blocked by 0.3% H_2_O_2_ in absolute methanol for 15 min at room temperature. The sections were then incubated overnight at 4 °C with anti-Nrf2 (Novus Biologicals, Littleton, CO, USA) and washed with PBS containing 0.05% Trion X-100. Incubation with a secondary antibody and the peroxidase–antiperoxidase (PAP) complex was carried out for 30 min at room temperature. Immunoreactive sites were visualized by 3,3′-DAB 3,3′-diaminobenzidine). Subsequently, the slices were counterstained by hematoxylin.

### 4.11. Statistical Analysis

All presented immunoblots are representative of the results obtained from at least three separate experiments. All grouped data are presented as mean ± SD. Differences between groups were analyzed by analysis of variance (ANOVA) or the Student’s t test, as appropriate, using the GraphPad Prism 7.0 software (GraphPad Software, Inc., La Jolla, CA, USA). All experiments were repeated in at least duplicate, with triplicate technical replicates performed.

## Supplementary Materials

The following are available online at https://www.mdpi.com/1422-0067/20/18/4366/s1. Figure S1: QC sensitizes CRC cells to 5-FU treatment under normoxia. Figure S2: QC sensitizes various cancer cells to 5-FU treatment under normoxia and hypoxia. Figure S3: Effects of Nrf2 expression in CRC cells. Figure S4: QC sensitizes CRC cells to 5-FU under hypoxia by inhibiting Nrf2.
