# Supplementary material for: Quinacrine-Mediated Inhibition of Nrf2 Reverses Hypoxia-Induced 5-Fluorouracil Resistance in Colorectal Cancer
Source: Int J Mol Sci. 2019 Sep 5;20(18):4366. doi: 10.3390/ijms20184366 (PMC6770959; doi:10.3390/ijms20184366)
Supplement: Supplementary file 1 [file ijms-20-04366-s001.pdf]

International Journal of *Molecular Sciences*

MDPI

Supplementary Materials

A

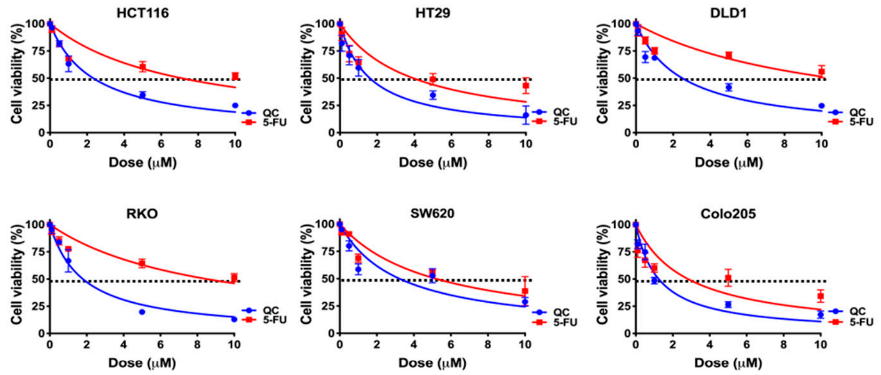

B

QC and 5-FU dose-response parameters for six colorectal cancer cell lines under normoxia.

	QC			5-FU		
Cell line	IC_{50} (μM)	95% CI	R^2	IC_{50} (μM)	95% CI	R^2
HCT116	2.334	2.103-2.708	0.9711	7.112	4.998-10.17	0.6223
HT29	1.607	1.216-2.131	0.9014	3.945	2.548-60.17	0.6478
DLD1	2.497	1.962-3.173	0.9196	10.60	7.921-14.43	0.6307
RKO	1.790	1.542-2.080	0.9748	8.512	6.782-10.75	0.8153
SW620	3.225	2.287-4.515	0.8315	5.187	3.977-6.670	0.8542
Colo205	1.237	1.010-1.519	0.9475	2.825	1.604-4.846	0.4749

C

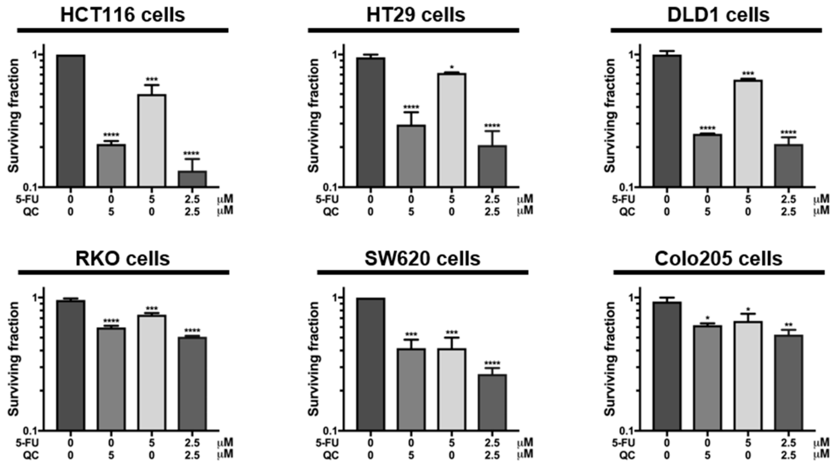

Figure 1. QC sensitizes CRC cells to 5-FU treatment under normoxia. (**A**) ATP-Glo assay for the QC or 5-FU treatment in HCT116, HT29, DLD1, RKO, SW620, Colo205 cells under normoxia. (**B**) the summary of IC_{50} of QC or 5-FU treatment of all the tested CRC cells is shown with 95% CI. (**C**) Clonogenic survival assay for the QC and 5-FU combination and single-agent treatment in HCT116, HT29, DLD1, RKO, SW620, and Colo205 cells.

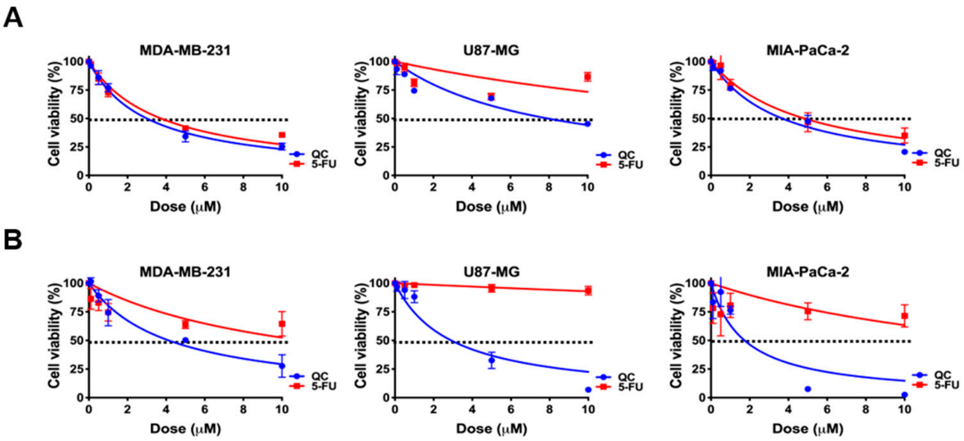

C

QC and 5-FU dose-response parameters for various cancer cell lines under normoxia and hypoxia.

Cell line		QC			5-FU		
		IC_{50} (μM)	95% CI	R^2	IC_{50} (μM)	95% CI	R^2
Normoxia	MDA-MB-231	3.003	2.698-3.342	0.9836	3.739	3.205-4.360	0.9600
	U87-MG	7.930	6.320-10.00	0.9071	27.55	17.44-50.69	0.5706
	MIA-PaCa-2	3.683	3.234-4.193	0.9742	4.821	3.969-5.859	0.9340
Hypoxia	MDA-MB-231	4.134	3.387-5.043	0.9379	11.02	7.164-17.65	0.1504
	U87-MG	2.910	2.243-3.775	0.9203	130.0	100.7-182.5	0.4716
	MIA-PaCa-2	1.731	1.209-2.479	0.9703	17.29	9.281-39.08	0.6641

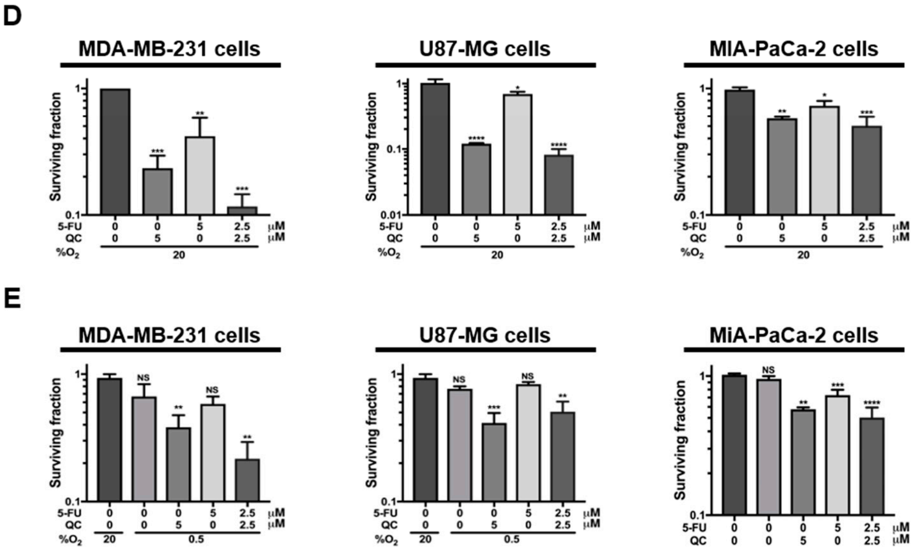

Figure 2. QC sensitizes various cancer cells to 5-FU treatment under normoxia and hypoxia. (**A**,**B**) ATP-Glo assay for the QC or 5-FU treatment in various cancer cells (MDA-MB-231, U87-MG, and MIA-PaCa-2) under normoxia (**A**) and hypoxia (**B**). (**C**) the summary of IC_{50} of QC or 5-FU treatment of all the tested various cancer cells is shown with 95% CI. (**D**,**E**) Clonogenic survival assay for the QC and 5-FU combination and single-agent treatment in various cancer cells under normoxia (**D**) and hypoxia (**E**).

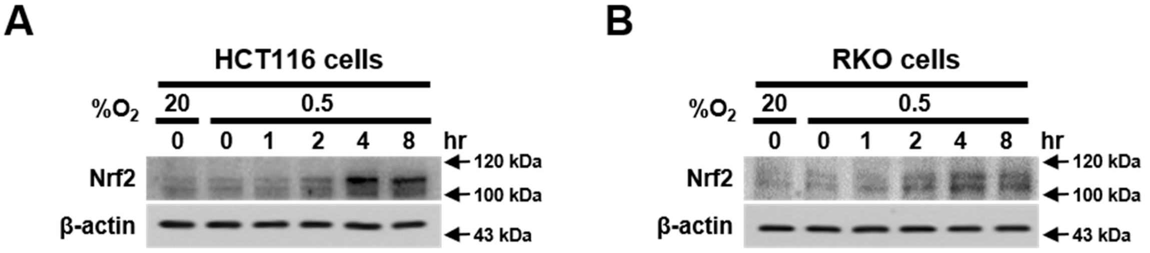

Figure 3. Effects of Nrf2 expression in CRC cells. (**A**,**B**) HCT116 (**A**) and RKO (**B**) cells were exposed to 0.5% O_2, and cells were harvested at the indicated times. Then, the whole-cell lysates were analyzed by immunoblotting for the indicated proteins. Representative images of Nrf2 and β-actin were detected by immunoblot.

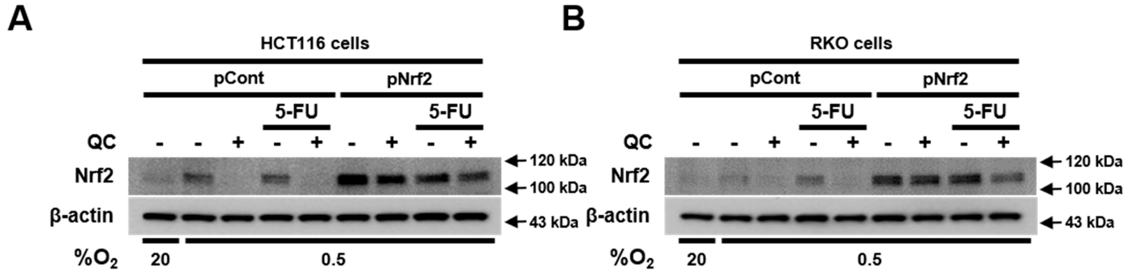

Figure 4. QC sensitizes CRC cells to 5-FU under hypoxia by inhibiting Nrf2. (**A**,**B**) HCT116 (**A**) and RKO (**B**) cells were transfected with or without pCont or pNrf2, treated with or without QC, 5-FU, or QC and 5-FU and exposed to 0.5% O_2. After 4 hr, the cells were harvested, and the whole-cell lysates were analyzed by immunoblotting for the indicated proteins. Representative images of Nrf2 and β-actin were detected by immunoblot.
